# Long-term cardiac pathology in individuals with mild initial COVID-19 illness

**DOI:** 10.1038/s41591-022-02000-0

**Published:** 2022-09-05

**Authors:** Valentina O. Puntmann, Simon Martin, Anastasia Shchendrygina, Jedrzej Hoffmann, Mame Madjiguène Ka, Eleni Giokoglu, Byambasuren Vanchin, Niels Holm, Argyro Karyou, Gerald S. Laux, Christophe Arendt, Philipp De Leuw, Kai Zacharowski, Yascha Khodamoradi, Maria J. G. T. Vehreschild, Gernot Rohde, Andreas M. Zeiher, Thomas J. Vogl, Carsten Schwenke, Eike Nagel

**Affiliations:** 1 Institute for Experimental and Translational Cardiovascular Imaging, DZHK Centre for Cardiovascular Imaging, Goethe University Frankfurt, University Hospital Frankfurt, Frankfurt am Main, Germany; 2 Department of Diagnostic and Interventional Radiology, University Hospital Frankfurt, Frankfurt am Main, Germany; 3 Institute of Cardiovascular Regeneration, Goethe University Frankfurt, Frankfurt am Main, Germany; 4 Infektiologikum, Frankfurt am Main, Germany; 5 Department of Anesthesiology, Intensive Care Medicine & Pain Therapy; Goethe University Frankfurt, University Hospital Frankfurt, Frankfurt am Main, Germany; 6 Department of Internal Medicine, Infectious Diseases, University Hospital Frankfurt, Goethe University Frankfurt, Frankfurt am Main, Germany; 7 Department of Internal Medicine, Respiratory Medicine, University Hospital Frankfurt, Goethe University Frankfurt, Frankfurt am Main, Germany; 8 SCO:SSiS Statistical Consulting, Berlin, Germany

**Keywords:** Heart failure, Viral infection, Diagnostic markers

## Abstract

Cardiac symptoms are increasingly recognized as late complications of severe acute respiratory syndrome coronavirus 2 (SARS-CoV-2) infection in previously well individuals with mild initial illness, but the underlying pathophysiology leading to long-term cardiac symptoms remains unclear. In this study, we conducted serial cardiac assessments in a selected population of individuals with Coronavirus Disease 2019 (COVID-19) with no previous cardiac disease or notable comorbidities by measuring blood biomarkers of heart injury or dysfunction and by performing magnetic resonance imaging. Baseline measurements from 346 individuals with COVID-19 (52% females) were obtained at a median of 109 days (interquartile range (IQR), 77–177 days) after infection, when 73% of participants reported cardiac symptoms, such as exertional dyspnea (62%), palpitations (28%), atypical chest pain (27%) and syncope (3%). Symptomatic individuals had higher heart rates and higher imaging values or contrast agent accumulation, denoting inflammatory cardiac involvement, compared to asymptomatic individuals. Structural heart disease or high levels of biomarkers of cardiac injury or dysfunction were rare in symptomatic individuals. At follow-up (329 days (IQR, 274–383 days) after infection), 57% of participants had persistent cardiac symptoms. Diffuse myocardial edema was more pronounced in participants who remained symptomatic at follow-up as compared to those who improved. Female gender and diffuse myocardial involvement on baseline imaging independently predicted the presence of cardiac symptoms at follow-up. Ongoing inflammatory cardiac involvement may, at least in part, explain the lingering cardiac symptoms in previously well individuals with mild initial COVID-19 illness.

## Abstract (web summary)

In individuals with long-term cardiac symptoms after an initially mild course of COVID-19 illness, magnetic resonance imaging and measurement of cardiac injury biomarkers commonly detected ongoing cardiac inflammation but not structural heart disease.

## Main

Lingering cardiac symptoms, including exercise intolerance, tachycardia and chest pain, are increasingly recognized late complications of COVID-19 (refs. ^1–6^). Myocardial injury, evidenced by elevated troponin, is common in hospitalized patients with pre-existing conditions and relates to the higher rates of cardiac complications and poor prognosis^7–9^. However, in home-isolated, non-hospitalized individuals with mild initial illness and no previous cardiac conditions, significant troponin rise is rarely found despite often profound symptoms. It remains uncertain whether persistent symptoms, at least in part, relate to cardiovascular involvement and what are the underlying pathophysiological correlates. Studies performed mainly in young athletic populations shortly after the initial infection recorded subtle myocardial inflammatory changes, non-ischemic myocardial scarring and pericarditis^10–13^.

To address the uncertainties of the pathophysiology and its relation to symptoms, we conducted serial cardiovascular assessments in a prospective study of selected individuals with no known heart conditions or significant comorbidities and mild initial COVID-19 illness using standardized questionnaires, blood sampling and cardiac magnetic resonance (CMR) imaging. Our main hypothesis was that there are differences in imaging and biomarker parameters between individuals with lingering cardiac symptoms after COVID compared to those without symptoms or to controls without previous infection. Additionally, we hypothesized that these markers improve at follow-up and that the symptoms at follow-up could be predicted from the baseline parameters.

## Results

### Overview of the study cohort

**Fig. 1:**
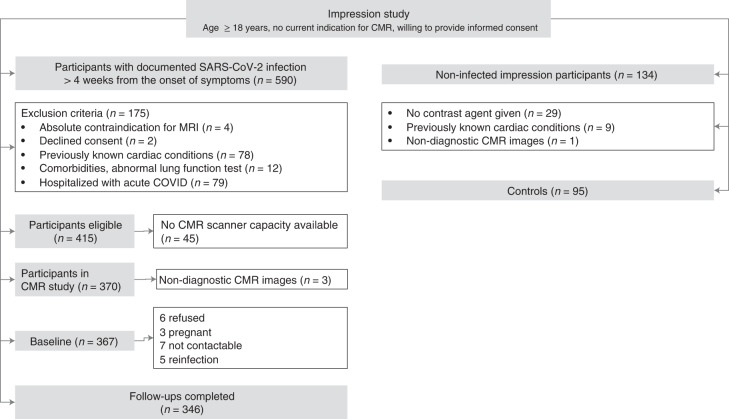
Study population flowchart. Number of participants eligible for inclusion at baseline and follow-up assessments. CMR: cardiovascular magnetic resonance.

This was a prospective, single-center, observational cohort study of individuals with possible subclinical cardiac involvement but no formal clinical indication for CMR imaging. In total, 346 individuals with prior COVID infection (hereafter referred to as ‘participants’) underwent a baseline scan after a minimum of 4 weeks from the diagnosis of initial COVID-19 between April 2020 and October 2021 and a follow-up examination after a minimum of 4 months from baseline (Fig. 1 and Extended Data Table 1). Participants were informed about the study via promotional material, disseminated via family practitioners, health authority centers, patient online groups and websites. All participants underwent systematic screening for eligibility before enrollment into the study, conducted by trained clinical research personnel using a standardized questionnaire. Individuals with prior cardiac diagnoses or significant comorbidities were not included. After successful completion of screening, an appointment for the baseline CMR imaging study was made. Given the uncertainties surrounding this new disease entity and a lack of standardized definitions for post-COVID symptoms, participants were included regardless of the presence of any cardiac symptoms at baseline, such as shortness of breath, chest pain, palpitations or syncope. Comparisons were made between participants with and without cardiac symptoms. In addition, comparisons were performed with a control group of individuals using a nested case–control study design (referred to as ‘controls’) with no previous COVID-19 infection and no known heart disease or comorbidities (*n* = 95), with a similar distribution for age, sex and cardiovascular risk factors.

### Baseline characteristics

**Extended Data Fig. 1:**
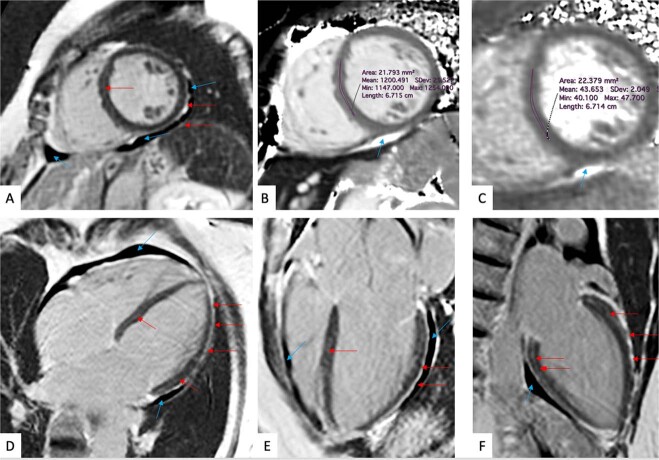
Representative magnetic resonance images of a symptomatic patient. (**a-f**) Late gadolinium enhancement imaging (A, D-F) and Native T1 (B) and T2 (C) mapping measurements of a 57-year-old woman evaluated 201 days after COVID-19 infection. This individual reported dyspnea, palpitations, and chest pain, worsening on minimal exertion. Late gadolinium enhancement imaging allows to visualize regional accumulation of the gadolinium-based contrast agent along the outer rim of the myocardial free wall (red arrows), as well as within the thickened pericardial layers, separated by small amounts of pericardial effusion (blue arrows).

Table 1 lists baseline demographic characteristics, blood results and imaging findings of the study cohort. The mean age of the study population was 43.3 years (s.d. ± 12.1; males 165, 48%). The median (IQR) time interval between the COVID-19 diagnosis and the baseline evaluation was 109 (77–177) days. At baseline, 252 (73% (68–77%)) participants reported cardiac symptoms not present before COVID-19 (Table 2). In most (130; 38% (35–43%)), cardiac symptoms were mild; 113 (33% (30–38%)) had moderate symptoms, whereas in nine (3% (1–5%)), the symptoms were severe, limiting the activities of daily life (ADLs). Exertional dyspnea was the most common cardiac symptom (*n* = 215, 62% (57–67%)), followed by palpitations (*n* = 96, 28% (23–33%)), atypical chest pain (*n* = 93, 27% (22–32%)) and syncope (*n* = 10, 3% (2–5%)). Extended Data Fig. 1 includes typical examples of CMR imaging findings. Compared to controls (Table 1), participants had significantly higher diastolic blood pressure (BP) (*P* = 0.001) and myocardial mapping values (*P* < 0.001). Participants had more non-ischemic myocardial scar by late gadolinium enhancement (LGE, *P* < 0.001), detectable pericardial effusion (*P* < 0.001) with no hemodynamic relevance and pericardial enhancement by gadolinium contrast uptake in the pericardial layers (*P* < 0.001) compared to controls. Compared to asymptomatic participants (Table 1), those with cardiac symptoms had significantly higher myocardial native T1 mapping values (*P* < 0.001). No differences were observed in blood biomarkers, including C-reactive protein (CRP), high-sensitivity troponin T (hs-TropT) and N-terminal pro-brain natriuretic peptide (NT-proBNP), either among participants or compared to controls. Structural heart disease by way of reduced left ventricular ejection fraction (LVEF) or reduced right ventricular ejection fraction (RVEF) or dilated heart cavity was observed only in a few participants.

**Table 1:** Baseline characteristics of the study cohort, including controls and all participants with COVID-19, with and without cardiac symptoms

Characteristic	Controls, *n* = 95	COVID-19, *n* = 346	*P* value	Asym, *n* = 94	Sym, *n* = 252	*P* value
Days from diagnosis		109 (77–177)	NA	104 (78, 150)	114 (77, 183)	1.00
Age (years)	40.4 ± 13.9	43.3 ± 12.1	0.04	43.2 ± 12.6	43.4 ± 11.9	0.89
Gender (male), *n* (%)	50 (53%)	165 (48%)	0.39	52 (55%)	113 (45%)	0.08
BMI (kg m^−2^)	24.9 ± 4.5	25.1 ± 4.3	0.69	25.0 ± 3.6	25.1 ± 4.6	0.89
Hypertension, *n* (%)	17 (18%)	50 (14%)	0.41	16 (17%)	34 (13%)	0.41
Diabetes, *n* (%)	3 (3.2%)	12 (3.5%)	1.00	4 (4.3%)	8 (3.2%)	0.74
Hypercholesterolemia, *n* (%)	19 (20%)	43 (12%)	0.06	16 (17%)	27 (11%)	0.11
Smoking, *n* (%)	12 (13%)	25 (7.2%)	0.09	6 (6.4%)	19 (7.5%)	0.71
Heart rate (bpm)	67 ± 9	70 ± 11	0.04	68 ± 10	71 ± 11	0.017
Systolic BP (mmHg)	127 ± 14	132 ± 17	0.04	133 ± 17	131 ± 16	0.27
Diastolic BP (mmHg)	79 ± 10	83 ± 11	0.001	82 ± 11	84 ± 11	0.20
CRP (mg dl^−1^)	0.1 (0.0, 0.2)	0.1 (0.0, 0.2)	0.86	0.1 (0.0, 0.2)	0.1 (0.0, 0.2)	0.48
hs-TropT (pg ml^−1^)	4.0 (3.0, 5.0)	4.1 (3.0, 5.8)	0.07	3.9 (3.0, 5.9)	4.1 (3.0, 5.7)	0.93
hs-TropT (by category)			0.07			0.21
Not detectable, *n* (%)	40 (43%)	105 (31%)		30 (33%)	75 (31%)	
Detectable, *n* (%)	53 (57%)	227 (67%)		59 (64%)	168 (69%)	
Abnormal, *n* (%)	0 (0%)	5 (1.5%)		3 (3.3%)	2 (0.8%)	
NT-proBNP (pg ml^−1^)	37.1 (20.5, 66.3)	43.5 (23.6, 78.6)	0.28	44.6 (23.9, 81.9)	43.2 (23.6, 77.6)	0.79
LVEF (%)	58.5 ± 4.0	56.6 ± 4.6	<0.001	56.1 ± 5.0	56.7 ± 4.4	0.24
LVEF (by category)			0.35			0.35
Normal, *n* (%)	95 (100%)	339 (98%)		90 (97%)	249 (99%)	
Reduced, *n* (%)	0 (0%)	6 (1.7%)		3 (3.2%)	3 (1.2%)	
GLS (%)	−21.2 ± 3.2	−19.4 ± 3.1	<0.001	−20.0 ± 3.3	−19.2 ± 3.0	0.13
LV-EDVi (ml m^−2^)	86.4 ± 13.6	86.3 ± 13.8	0.96	89.2 ± 14.9	85.2 ± 13.3	0.017
LV-EDVi (by category)			1.00			0.50
Normal, *n* (%)	92 (97%)	335 (97%)		90 (96%)	245 (97%)	
Dilated, *n* (%)	3 (3.2%)	11 (3.2%)		4 (4.3%)	7 (2.8%)	
LV mass (ml m^−2^)	49.7 ± 9.5	47.0 ± 9.2	0.01	48.4 ± 10.4	46.4 ± 8.6	0.068
RVEF (%)	56.0 ± 5.8	54.0 ± 5.6	0.002	53.1 ± 5.9	54.4 ± 5.4	0.061
RVEF (by category)			0.59			0.13
Normal, *n* (%)	95 (100%)	341 (99%)		91 (97%)	250 (99%)	
Reduced, *n* (%)	0 (0%)	5 (1.4%)		3 (3.2%)	2 (0.8%)	
Native T1 (ms)	1,103 ± 37	1,129 ± 34	<0.001	1,116 ± 35	1,134 ± 33	<0.001
Native T2 (ms)	36.1 ± 1.7	38.3 ± 1.7	<0.001	37.8 ± 1.3	38.5 ± 1.7	<0.001
LGE (any)	7 (7.4%)	135 (39%)	<0.001	34 (36%)	101 (40%)	0.51
LGE (by type)			<0.001			0.35
None, *n* (%)	87 (92%)	208 (60%)		58 (62%)	150 (60%)	
Ischemic, *n* (%)	1 (1.1%)	3 (0.9%)		2 (2.1%)	1 (0.4%)	
Non-ischemic, *n* (%)	7 (7.4%)	132 (38%)		33 (35%)	99 (39%)	
Both, ischemic and non-ischemic, *n* (%)	0 (0%)	3 (0.9%)		1 (1.1%)	2 (0.8%)	
Pericardial effusion (any detectable), *n* (%)	32 (34%)	251 (73%)	<0.001	64 (68%)	187 (74%)	0.26
Pericardial effusion (by size), *n* (%)			<0.001			0.18
No	63 (66%)	95 (27%)		30 (32%)	65 (26%)	
<1 cm	32 (34%)	238 (69%)		63 (67%)	175 (69%)	
≥1 cm	0 (0%)	13 (3.8%)		1 (1.1%)	12 (4.8%)	
Pericardial enhancement, *n* (%)	15 (16%)	161 (47%)	<0.001	33 (35%)	128 (51%)	0.009

Asym, asymptomatic symptom status; BMI, body mass index; LV- EDVi, left ventricular end-diastolic volume, indexed to body surface area; Sym, symptomatic symptom status. Ischemic and non-ischemic scar may occur in the same patient. The categorical classifications of LVEF, RVEF and LV-EDVi are based on the UK Biobank study^30^.

**Table 2:** Summary of cardiac symptoms

Characteristic	Controls, *n* = 95	COVID-19, *n* = 346	*P* value	Sym (baseline), *n* = 252	Sym (follow-up), *n* = 252	*P* value
Shortness of breath			<0.001			<0.001
None, *n* (%)	81 (85%)	131 (38%)		37 (15%)	99 (39%)	
With strenuous activities, *n* (%)	12 (13%)	121 (35%)		121 (48%)	87 (35%)	
During daily activities, *n* (%)	2 (2.1%)	87 (25%)		87 (35%)	61 (24%)	
At rest, *n* (%)	0 (0%)	7 (2.0%)		7 (2.8%)	5 (2.0%)	
Palpitations, *n* (%)	17 (18%)	96 (28%)	0.069	96 (38%)	77 (31%)	0.09
Chest pain			0.002			0.09
None, *n* (%)	88 (93%)	267 (77%)		173 (69%)	193 (77%)	
With strenuous activities, *n* (%)	4 (4.2%)	24 (6.9%)		24 (9.5%)	11 (4.4%)	
During daily activities, *n* (%)	3 (3.2%)	15 (4.3%)		15 (6.0%)	13 (5.2%)	
At rest, *n* (%)	0 (0%)	40 (12%)		40 (16%)	35 (14%)	
Syncope, *n* (%)	0 (0%)	10 (2.9%)	0.13	10 (4.0%)	6 (2.4%)	0.4
PCFS			<0.001			<0.001
None, *n* (%)	65 (68%)	94 (27%)		0 (0%)	70 (28%)	
Mild, *n* (%)	27 (28%)	130 (38%)		130 (52%)	88 (35%)	
Moderate, *n* (%)	3 (3.2%)	113 (33%)		113 (45%)	87 (35%)	
Severe, *n* (%)	0 (0%)	9 (2.6%)		9 (3.6%)	7 (2.8%)	

Left side: reported by controls and all patients with COVID-19; right side: reported by symptomatic participants with COVID-19 at baseline and follow-up. Asymptomatic participants were not included. Sym, symptomatic participants with COVID-19.

### Follow-up assessments

Consecutive follow-up assessments were performed after a median of 329 days (274–383) from COVID-19 diagnosis. Cardiac symptoms were present in 198 (57% (52–62%)) participants at follow-up; symptoms persisted in a total of 182 (53% (47–58%), Sym/Sym) participants; 16 (5% (3–7%)) previously asymptomatic participants developed new symptoms (Asy/Sym); 78 (23% (18–27%) Asy/Asy) originally asymptomatic participants remained asymptomatic; and 70 (20% (18–27%), Sym/Asy) participants became asymptomatic. Women were more likely to continue experiencing cardiac symptoms than men (*n* = 122, 67% (61–74%) versus *n* = 76, 46% (39–54%); *P* < 0.001). In the overall cohort (Table 3), there was a trend for reduction of heart rate (*P* = 0.006), systolic blood pressure (BP) (*P* = 0.006) and native T1 (*P* = 0.003) and a significant reduction of T2 (*P* < 0.001) and increase of RVEF (*P* < 0.001). Extended Data Table 2 provides patient characteristics based on symptom status at baseline/follow-up. In participants with persistent symptoms at follow-up (Sym/Sym), native T2 showed a trend toward higher values compared to participants who became asymptomatic (Sym/Asy) (least square means, adjusted *P* = 0.04; Table 4). The predictive associations of baseline parameters for symptomatic status at follow-up are shown in Table 5. In univariate analyses, female gender, small LV volumes and mass and native T1 at baseline were associated with symptomatic status at follow-up. In the final multivariate model, female gender and increased native T1 at baseline independently predicted the presence of cardiac symptoms at follow-up.

**Table 3:** Characteristics of the COVID-19 cohort at baseline and follow-up

Characteristic	Baseline *n* = 346	Follow-up *n* = 346	Adjusted difference	95% CI	*P* value
Days from diagnosis	109 (77, 177)	329 (274, 383)			
Heart rate (bpm)	70 ± 11	68 ± 11	1.7	0.49, 2.8	0.006
Systolic BP (mmHg)	132 ± 17	128 ± 17	2.5	0.72, 4.2	0.006
Diastolic BP (mmHg)	83 ± 11	82 ± 12	0.62	−0.60, 1.9	0.318
CRP (mg dl^−1^)	0.1 (0.0, 0.2)	0.1 (0.0, 0.2)	−0.16	−0.46, 0.13	0.280
hs-TropT (pg ml^−1^)	4.1 (3.0, 5.8)	3.6 (3.0, 5.7)	0.09	−0.20, 0.37	0.550
NT-proBNP (pg ml^−1^)	43.5 (23.6, 78.6)	41.0 (21.0, 75.0)	0.73	−3.9, 5.4	0.757
LVEF (%)	56.6 ± 4.6	56.9 ± 4.8	−0.25	−0.75, 0.24	0.318
GLS (%)	−19.4 ± 3.1	−18.7 ± 3.2	−0.44	−0.92, 0.04	0.070
LV-EDVi (ml m^−2^)	86.3 ± 13.8	85.2 ± 13.9	0.75	−0.72, 2.2	0.315
LV mass index (g m^−2^)	47.0 ± 9.2	47.5 ± 9.2	−0.39	−1.4, 0.66	0.45
RVEF (%)	54.0 ± 5.6	55.4 ± 5.6	−1.0	−1.6, −0.39	0.001
Native T1 (ms)	1129 ± 34	1122 ± 28	5.0	1.7, 8.3	0.003
Native T2 (ms)	38.3 ± 1.7	37.7 ± 1.3	0.42	0.26, 0.58	<0.001

LV- EDVi, left ventricular end-diastolic volume, indexed to body surface area. The categorical classifications of LVEF, RVEF and LV-EDVi are based on the UK Biobank study^30^.

**Table 4:** Least square means analysis of follow-up findings, adjusted for baseline values

Symptom status (baseline/follow-up)	Sym/Sym	Sym/Asym	Asym/Sym	Asym/Asym	*P* value
	*n* = 182	*n* = 70	*n* = 16	*n* = 78	
Heart rate (bpm)	68.2 (66.8–69.5)	65.8 (63.6–67.9)	67.7 (63.2–72.2)	67.2 (65.1–69.3)	0.31
Systolic BP (mmHg)	127 (125–129)	127 (123–130)	130 (123–137)	132 (129–135)	0.04
LVEF (%)	57.0 (56.4–57.5)	56.6 (55.7–57.5)	57.6 (55.7–59.5)	57.0 (56.2–57.9)	0.8
GLS (%)	–18.9 (−19.6 to −18.2)	−18.5 (−19.8 to −17.3)	−19.9 (−22.3 to −17.4)	−19.2 (−20.4 to −18.0)	0.75
LV-EDVi (ml m^−2^)	84.0 (82.7–85.2)	84.1 (82.1–86.1)	87.4 (83.3–91.5)	86.4 (84.5–88.3)	0.10
LV mass index (g m^−2^)	46.5 (45.3–47.7)	47.9 (46.0–49.8)	48.0 (44.0–51.9)	48.7 (46.9–50.5)	0.22
RVEF (%)	55.9 (55.2–56.7)	54.3 (53.1–55.5)	56.0 (53.5–58.4)	55.1 (53.9–56.2)	0.14
Native T1 (ms)	1,124 (1,121–1,127)	1,119 (1,114–1,124)	1,119 (1,107–1,129)	1,119 (1,114–1,124)	0.27
Native T2 (ms)	37.9 (37.7–38.0)	37.4 (37.2–37.7)	38.0 (37.5–38.5)	37.5 (37.3–37.8)	0.01

ANCOVA was used to compare among all groups after adjustment for baseline differences. Asym, asymptomatic symptom status; LV- EDVi, left ventricular end-diastolic volume, indexed to body surface area; Sym, symptomatic symptoms status. Symptom status is shown at baseline and at follow-up (baseline/follow-up).

**Table 5:** Baseline factors associated with symptoms at follow-up

	Univariate			Multivariate		
Characteristic	OR^1^	95% CI^1^	*P* value	OR^1^	95% CI^1^	*P* value
Age (years)	1.02	1.00, 1.04	0.032	–	–	–
Gender (female)	2.42	1.57, 3.76	<0.001	2.26	1.42, 3.63	<0.001
Heart rate (bpm)	1.02	1.00, 1.04	0.036	–	–	–
Systolic BP (mmHg)	1.00	0.98, 1.01	0.447	–	–	–
Diastolic BP (mmHg)	1.01	0.99, 1.03	0.188	–	–	–
CRP (mg dl^−1^)	1.36	0.47, 4.22	0.571	–	–	–
hs-TropT (pg ml^−1^)	0.97	0.88, 1.06	0.452	–	–	–
NT-proBNP (pg ml^−1^)	1.00	1.00, 1.01	0.128	–	–	–
LVEF (%)	1.03	0.98, 1.08	0.231	–	–	–
GLS (%)	0.99	0.90, 1.09	0.904	–	–	–
LV- EDVi (ml m^−2^)	0.96	0.95, 0.98	<0.001	–	–	–
LV mass index (g m^−2^)	0.96	0.94, 0.98	<0.001	–	–	–
RVEF (%)	1.06	1.02, 1.10	0.003	–	–	–
Native T1 (10 ms)	1.14	1.07, 1.20	<0.001	1.13	1.06, 1.20	<0.001
Native T2 (ms)	1.22	1.07, 1.40	0.003			
LGE (non-ischemic), present	1.72	1.10, 2.70	0.016			
Pericardial effusion, present	1.38	0.86, 2.23	0.18			
Pericardial enhancement	1.39	0.90, 2.14	0.134			
Time from diagnosis	1.00	1.00, 1.00	0.37			

Univariate and multivariate logistic regression analyses are shown.

## Discussion

In this cohort of selected previously well and home-isolating individuals initially evaluated at a median of 109 days and followed-up at 329 days after diagnosis of COVID-19 infection, respectively, the ongoing cardiac symptoms were related to mild but notable imaging findings, suggesting inflammatory cardiac involvement. At baseline, participants with cardiac symptoms had higher mapping values, suggesting diffuse myocardial inflammation, and more frequent pericardial enhancement, suggesting pericardial inflammatory involvement compared to asymptomatic participants. At follow-up, 53% of the cohort had persistent cardiac symptoms, whereas new symptoms developed in 5%. Mapping values improved in all participants but showed a trend toward higher values in those with persistent cardiac symptoms versus those who became asymptomatic. Female gender and higher baseline native T1 predicted the symptomatic status at follow-up. Our findings suggest that persistent cardiac symptoms in previously well, home-isolated individuals may, at least in part, relate to mild chronic inflammatory cardiac involvement in the absence of significant structural heart disease or increased levels of cardiac biomarkers.

Notably, as our study focused on a selected population of individuals with prior COVID illness, it does not inform on the prevalence of cardiac symptoms after COVID. However, it provides important insights into their spectrum and subsequent evolution.

Symptoms after COVID infection are common and wide-ranging and often include cardiac symptoms, such as exertional dyspnea and chest pains. In the COVID Symptoms Study, cardiac symptoms amounted to 6.1% of participants with persistent symptoms^5^. In a group of previously hospitalized patients, persistent symptoms were reported in 72%, with dyspnea featuring as the most prevalent symptom (54.8 %)^14^. Other investigators found non-cardiac symptoms, including fatigue, muscle weakness and sleep difficulties, more prevalent after 6 months, whereas palpitations and chest pain were described by 9% and 5 % of patients, respectively^15^. A more recent review of hospital records and health outcomes in survivors of COVID-19 beyond 30 days indicates higher mortality, rate of hospitalizations and outpatient visits, including for cardiovascular issues, such as hypertension, arrhythmias, chest tightness and heart failure^8^.

The findings of the current study expand on these previous reports by providing a focused assessment of non-hospitalized individuals with no previous comorbidities, thus allowing a more direct insight into post-infection sequelae. This was purposeful to avoid mixing with the effects of severe disease and the pathophysiology accrued during the severe initial infection. Individuals who require hospitalization are also more likely to have pre-existing heart conditions. We further excluded individuals with abnormal lung function tests to avoid confounding the underlying cause of breathlessness. We demonstrate that, in the present cohort, exertional dyspnea was the most frequently experienced cardiac symptom. Based on the clinical interviews, this was manifested as a wide spectrum of exercise intolerance, from the inability to regain a previous level of fitness, climbing stairs or attempting inclines, to the limiting physical aspects of professional or everyday life. Shortness of breath was often linked to an exaggerated tachycardia response and post-exertional fatigue. The more affected participants refrained from leaving their homes due to sudden onset of general physical weakness, dizziness or even blackouts. Although not assessed formally or in a quantitative way in the present study, the observed patterns of symptoms may guide future research. In our cohort, cardiac symptoms were new and not present before COVID-19 infection. They were also not explained by any pre-existing conditions, such as those of cardiac or respiratory origin or by the consequences of severe initial COVID-19 illness, given the exclusion criteria including hospitalization or abnormal lung function tests after the initial illness.

The results of our study may shed light on the underlying pathophysiological mechanisms contributing to cardiac symptoms. We employed CMR imaging techniques to detect diffuse inflammatory myocardial involvement by T1 mapping, which is a non-specific measure of abnormal myocardium, and T2 mapping, which mainly relates to myocardial water content. LGE denotes regional myocardial injury due to the accumulation of the gadolinium-based contrast agent. Accumulation of gadolinium contrast agent also helps to visualize thickened pericardial layers, separated by small amounts of pericardial effusion. In this study, we first found signs of inflammatory myopericardial involvement, which persisted several months after the initial COVID illness. At baseline, participants showed higher myocardial mapping measurements, more pericardial involvement and more non-ischemic scar, but not higher troponin and NT-proBNP levels, compared to controls. Second, these findings were found to be significantly more pronounced in participants with cardiac symptoms. Although the magnitude of these changes was generally mild and not associated with profound structural heart disease, we were able to show improvement at follow-up. The reduction of myocardial mapping values was detectable across the whole cohort; however, native T2 remained higher in the subgroup with persistent cardiac symptoms. Thus, the imaging findings suggest that inflammatory cardiac involvement after COVID may be a pathophysiological commonality shared among all individuals, regardless of the expression of cardiac symptoms. The underlying pathological mechanism for the detected increase in myocardial water content remains unclear at this stage and may relate to changes in vascular, cellular or interstitial permeability, but it is unlikely explained by direct myocyte injury/necrosis, as thought to be the core mechanism in classical viral myocarditis^16^. The absence of myocardial necrosis may also explain the absence of profoundly reduced ejection fraction or dilated heart cavities in this previously healthy population that had not sustained a considerable cardiac or pulmonary injury during the initial illness^6^. Even so, there was a trend toward a small reduction of LV and RV function and global longitudinal strain (GLS) in participants compared to controls and an improvement of RVEF at follow-up. Although 64% of participants had detectable troponin, the overall levels of troponin were low and not related to the presence of cardiac symptoms, reiterating the suggestion of increased myocardial wall stress and not necrosis as a likely mechanism of symptoms. The observation of higher systolic BP and resting heart rate in a previously prevalently non-hypertensive cohort are interesting and suggest an underlying mechanism of an increase of vascular stiffness, adding to the afterload^17–19^, while coupled with myocardial edema, further hindering the efficiency of diastolic filling^19–21^. The non-ischemic LGE pattern resembles the changes frequently observed in chronic systemic inflammatory conditions^22^, where increased vascular stiffness is well-established^23^. The latter conditions tend to be more frequent in females, potentially explaining the higher proportion of symptomatic females after COVID^24,25^. Small-vessel endothelial dysfunction and microthrombosis could explain the few cases with ischemic subendocardial LGE pattern, which is otherwise more likely associated with coronary artery disease^26^. The present findings require confirmation in future studies, including determination of the long-term or prognostic relevance, if any.

Several limitations apply to this study. First, this is a proof-of-concept, single-center cohort study of cardiovascular pathophysiology with imaging techniques that are not widely available. Although mapping techniques provide valuable pathophysiological insights, this information is not immediately transferable into clinical practice owing to a lack of standardization and methodological variations. We have not deployed endomyocardial biopsy or Lake Louise criteria in determining myocardial inflammation owing to their low sensitivity for diffuse disease and reliance on areas of considerable necrosis^27,28^. Also, existing literature excludes myocarditis with viral replication as the prevalent mechanism of post-COVID injury^10,29^. This was an exploratory study in a new disease entity, and the effect size was unknown; as such, an a priori sample size estimation was not possible. The results of the comparison of the four subgroups based on their symptom status at baseline and follow-up should be regarded of exploratory nature, which require confirmation in larger samples, if available. Assessment of the pericardial space was not pre-specified in the study protocol, as this observation was made only in the context of the new disease. Selection bias due to recruitment by self-referral cannot be excluded. Although we included only participants with no indication for CMR imaging and applied the same exclusion criteria for the COVID population and the control group, there may be some remaining unrecognized differences. Owing to the limited number of available controls, no matching was performed. However, controls had a similar distribution of age, sex and cardiovascular risk factors. Also, significances are not regarded as confirmatory but as hypothesis-generating, as only timely relationships, but no causal relationship, can be assessed in such type of study. The effect of vaccination was not systematically assessed. In total, 144 participants received an mRNA vaccination between the baseline and the follow-up scan. We performed separate analyses for participants with vaccination as well as for participants without vaccination. The results were not different from the findings of the full cohort as presented. The cardiac effects of vaccination require further research.

In summary, in the present cohort of individuals with mild initial COVID-19 illness, cardiac symptoms were related to subclinical inflammatory cardiac involvement, which may, at least in part, explain the pathophysiological background of persistent cardiac symptoms. Notably, profound myocardial injury or structural heart disease is not prerequisite for the presence of symptoms defying the classical definitions of viral myocarditis. Subclinical cardiovascular inflammation is increasingly recognized as a risk factor in chronic autoimmune systemic conditions, necessitating further research to establish long-term outcome in the context of post-COVID.

## Methods

### Ethical considerations

All participants provided written informed consent. The study was approved by the institutional ethics committee (Ethics Committee of University Hospital Frankfurt of Goethe University; impression study, NCT04444128; https://www.cardiac-imaging.org/covid19—heart-study.html). All procedures were performed in concordance with the Declaration of Helsinki and the International Conference on Harmonization of Good Clinical Practice. No compensation was provided to participants.

### Study population

This is a prospective, single-center, observational cohort study of individuals with possible subclinical cardiac involvement but no formal clinical indication for CMR imaging. Consecutive participants with confirmatory laboratory evidence of SARS-CoV-2 infection by detection of ribonucleic acid in a swab test of the upper respiratory tract using an approved method were recruited^31^. Participants were informed about the study via promotional material, disseminated via family practitioners, health authority centers, patient online groups and websites. All participants underwent systematic screening for eligibility before enrollment into the study, conducted by trained clinical research personnel using a standardized questionnaire. Individuals with prior cardiac diagnoses or significant comorbidities were not included. Only upon successful completion of screening, an appointment for the baseline CMR imaging study was made. The catchment area included the German states of Hesse, Rhineland-Pfalz and Baden-Wurttemberg, primarily concentrated around the cities of Frankfurt, Darmstadt, Wiesbaden and Mainz. The recruitment commenced in April 2020.

Exclusion criteria were:

1. Hospitalized during the acute COVID illness;
2. An established diagnosis of cardiovascular disease (defined as a previous diagnosis of hemodynamically significant coronary artery disease^32^, history of revascularization or cardiovascular device implantation or structural heart disease, heart failure, cardiomyopathy, significant valvular disease (grade 3 or higher), congenital heart disease, peripheral artery disease, etc.);
3. Known pre-existing significant lung conditions^33,34^ or persistently abnormal lung function test after recovery from the acute infection;
4. Individuals with known liver or kidney disease, uncontrolled diabetes or other significant endocrine, rheumatological or oncological conditions; clinically and laboratory euthyroid individuals receiving thyroxine supplements were not excluded;
5. Individuals with known absolute contraindications to MRI;
6. Individuals unwilling to participate in long-term follow-ups.

The participants represent individuals who contracted SARS-CoV-2 in the community and did not require admission to the hospital during the acute illness. Due to uncertainty surrounding this new clinical entity, the presence of any post-COVID symptoms at the time of recruitment was not mandatory for inclusion. A total of 58 patients were included in a previous publication^10^.

Comparisons were made with a control group, recruited from within a prospective cohort study using a nested case–control study design. The control group was similar in terms of age, sex and cardiovascular risk factors and had no known previous heart disease or comorbidities (Fig. 1). CMR imaging findings were not used to pre-select any of these individuals. Members of the control group who opted out from receiving gadolinium contrast agent were not included. For controls included after March 2020, negative COVID status was ascertained through history and serological testing.

### Clinical data

All consenting participants attended a baseline visit. In line with the focus on the late consequences, the baseline visit was scheduled after a minimum of 4 weeks from diagnosis of the acute COVID-19 illness. In total, 346 consecutive participants completed a follow-up visit after a minimum of 4 months from the baseline visit. At each visit, demographic characteristics, risk factors, symptoms, resting blood pressure and heart rate, blood sampling and MRI were performed in all participants.

### Assessment of cardiac symptoms

Symptoms were recorded at each visit and classified as cardiac and non-cardiac. Chest pain, dyspnea, palpitations and syncope were considered cardiac symptoms, which were recorded and graded using standardized questionnaires. Chest discomfort (Extended Data Table 3) was graded using a modified Chest Discomfort Scale based on the Canadian Chest Pain Scale^35^, not limited to the typical anginal type of chest pain, but also included, deep, dull, pulling, burning or sharp chest discomfort or tightness, radiating into the neck, back, shoulders or arms. To differentiate this from precordial catch symptoms and abdominal stiches, the criterion for chest pain had to last more than 10 minutes to qualify.

Dyspnea was graded using modified Medical Research Council Dyspnea Severity Score^36^ (Extended Data Table 4). Palpitations and syncope were classified as present or absent. The severity of symptoms was classified using a four-category ordinal grading scale (grade 1: none; grade 2: mild (with strenuous activity); grade 3: moderate (during ADLs); and grade 4: severe (present at rest)). The overall severity symptomatic class was defined by the highest grade of any of the above cardiac symptoms. Non-cardiac symptoms were recorded as a free text entry. Functional status, including the ADLs, were assessed using the Post-COVID-19 Functional Status (PCFS) scale^37^ (Extended Data Table 5). The PCFS scale is different from the other classifications used, as it describes severity rather than qualifying the symptoms. As such, a patient can have chest pain at rest but still be in a low PCFS class if the pain is not disabling.

### Laboratory methods

Participants were asked to present the confirmatory evidence of SARS-CoV-2 infection by an approved method by a certified laboratory for either reverse transcription–polymerase chain reaction for detection of viral ribonucleic acid on a swab test of the upper respiratory tract or clinical diagnosis with a subsequent supportive laboratory serological evidence of a specific antibody test (*n* = 6)^25^. Blood sampling for cardiac biomarkers was performed immediately before the CMR imaging study. Blood samples were processed in a local laboratory using standardized commercially available test kits for analysis of high-sensitivity C-reactive protein (hs-CRP), hs-TropT and NT-proBNP (Elecsys 2010, Roche). The local laboratory cutoff value for a limit of detection of hs-TropT was greater than 3 pg ml^−1^ and greater than 13.9 pg ml^−1^ for a significant increase above the 99th percentile^38^.

### Image acquisition and post-processing

All participants underwent a standardized CMR imaging protocol on a 3T scanner equipped with advanced cardiac software and a multi-channel coil (Skyra and Prisma, Siemens Healthineers, software version VE11). Scanner maintenance, calibration and quality assurance procedures were regularly provided by the vendor.

At baseline, acquisition of cardiac function, volumes, mass, myocardial mapping and scar imaging was performed. Follow-up scans included cardiac function, volumes, mass and myocardial mapping (non-contrast scans). Optional scans, such as T1-weighted and T2-weighted imaging, or post-contrast mapping were not performed^39^. Imaging parameters and scanning and shimming procedures for all sequences were standardized and mandatorily performed by all operators in all scans. Comparability and reproducibility of measurements were determined at each location. Relevant imaging parameters are included below^10,40^. Slice thickness in all acquisitions was set uniformly at 8 mm.

All participants were instructed about the importance of optimal breath-holding during the scanning and specifically for mapping and LGE acquisitions. All scanning protocols were optimized to enable maximal participant collaboration. All acquired images were routinely examined for quality during data acquisition, especially the quality of the mapping images during the acquisition, for artifacts and their influence on the measurements. All staff conducting the image acquisition underwent extensive training in all CMR scanning procedures with regular quality control assessments to enable the required level of standardization and image quality.

Cine imaging was performed using a balanced steady-state free precession sequence in combination with parallel imaging (GRAPPA) and retrospective gating during expiratory breath-hold (TE/TR/flip-angle: 1.7 ms/3.4 ms/30°; spatial resolution, 1.8 × 1.8 ×8 mm), as a short-axis (SAX) stack for assessment of cardiac volumes and function or single-slice long-axis views (two-chamber, three-chamber and four-chamber views).

Myocardial T1 and T2 mapping were acquired using sequences, which were validated histologically, clinically and against outcome^41–43^. For T1 mapping, a locally modified version of balanced steady-state free precession single breath-hold-modified Look-Locker imaging was performed in a single midventricular SAX slice at mid-diastole, before contrast administration, respectively (TE/TR/flip angle: 1.64 ms/3.3 ms/50°; acquired voxel size, 1.3 × 1.3 ×8 mm or smaller; phase-encoding steps, *n* = 166, 6/8 half scan, 11 images corresponding to three different inversion times using a non-selective 180° pre-pulse in an algorithm of n-images/n-beats 3b(2b)3b(2b)5b MOLLI scheme).

T2 mapping was performed using a T2 gradient echo single-shot FLASH sequence to generate three T2-weighted images with different T2 preparation times (T2P = 0 ms, 30 ms and 55 ms). Other sequence parameters included repetition time 3 × RR, acquisition time 7 × RR, echo time 1.32 ms, flip angle 12°, bandwidth 1,185, parallel acquisition (acceleration) GRAPPA 2 and acquired voxel size of 1.3 × 1.3 ×8 mm or smaller.

LGE was performed using whole heart coverage of SAX and LAX slices ~10 minutes after administration of 0.1 mmol gadobutrol per kilogram of body weight (Gadovist, Bayer), using a mid-diastolic inversion prepared two-dimensional gradient echo sequence (TE/TR/flip angle: 2.0 ms/3.4 ms/20°; acquired voxel size minimum, 1.4 × 1.4 ×8 mm) with an individually adapted pre-pulse delay achieving optimally nulled myocardium. Magnitude and phase-sensitive image reconstructions were evaluated.

Cardiac volumes, function and mass were measured in line with standardized post-processing recommendations^44^. Papillary muscles were included as a part of the LV blood volume (suiteHEART, NeoSoft). LV end-diastolic volumes (EDVs) and end-systolic volumes (ESVs) were determined using the rule of discs. Ejection fraction was computed as (EDV − ESV) / EDV. All volumetric indices were normalized to body surface area. Reference values were based on the UK Biobank Study^30^. Myocardial T1 and T2 relaxation times were measured in the septal myocardium of the midventricular SAX slice^45^, as per internal standardized operating procedures and with quality control by the core laboratory staff, blinded to the underlying clinical information and group allocation using pseudonymized datasets. The quality of the motion correction and co-registration of the inline maps was verified on the scanner, and, if unsatisfactory, the acquisition was repeated. Manual motion correction by propagating the septal region of interest through the individual images, followed by the manual image co-registration, was performed if not resolved by the means above. Areas of LGE were excluded from the measurements to avoid confounding diffuse fibrosis with replacement scar. Interpretation of LGE images followed standardized post-processing recommendations. Myocardial LGE was visually defined by two observers based on the presence and predominant pattern as ischemic or non-ischemic^44^. Pericardial involvement was considered present when enhancement involved both pericardial layers, irrespective of the size of pericardial effusion^46^. Cine images were employed for derivation of GLS analyses using Medis Suite MR version 2.1 (Medis Medical Imaging Systems)^47^.

### Statistical analysis

Data were entered using electronic case report forms in REDCap (Research Electronic Data Capture, Vanderbilt University). All analysis was performed using R version 3.6.1 (https://www.r-project.org/). Normality of distributions was tested using the Shapiro–Wilk test. Categorical data are presented as counts (percentages) and continuous variables as mean (± s.d.) or medians (IQRs), as appropriate for the type of data. Comparisons between groups were conducted using one-way ANOVA for normally distributed parameters and Kruskal–Wallis rank-sum test for non-normally distributed data. Fischer exact and χ^2^ tests were used for proportions. ANCOVAs adjusted for baseline values were used to assess the differences between the baseline and follow-up of the whole cohort as well as to compare follow-up observations between different groups and timepoints, using the baseline variable as a covariate. Least square means with 95% confidence intervals (CIs) were reported with adjustment for baseline. In case of *P* < 0.05 on the global level, pairwise comparisons were conducted and adjusted using Bonferroni correction. Exploratory univariate and multivariate analyses were used to predict the presence of symptoms at follow-up using binary logistic regression and presented with odds ratios (ORs), 95% CIs and *P* values. *q*-values are provided to correct for the false discovery rate in multiple testing. The univariate analysis focuses on the potential association of clinical, blood and imaging variables with regard to cardiac symptom status as the outcome variable. We included a priori variables of interest but omitted rarer occurrences, such as risk factors. All tests were two-tailed. *P* values less than 0.001 were considered statistically significant.

### Reporting summary

Further information on research design is available in the Nature Research Reporting Summary linked to this article.

## Online content

Any methods, additional references, Nature Research reporting summaries, source data, extended data, supplementary information, acknowledgements, peer review information; details of author contributions and competing interests; and statements of data and code availability are available at 10.1038/s41591-022-02000-0.

### Supplementary information

Reporting Summary

## Figures and Tables

**Extended Data Table 1:**
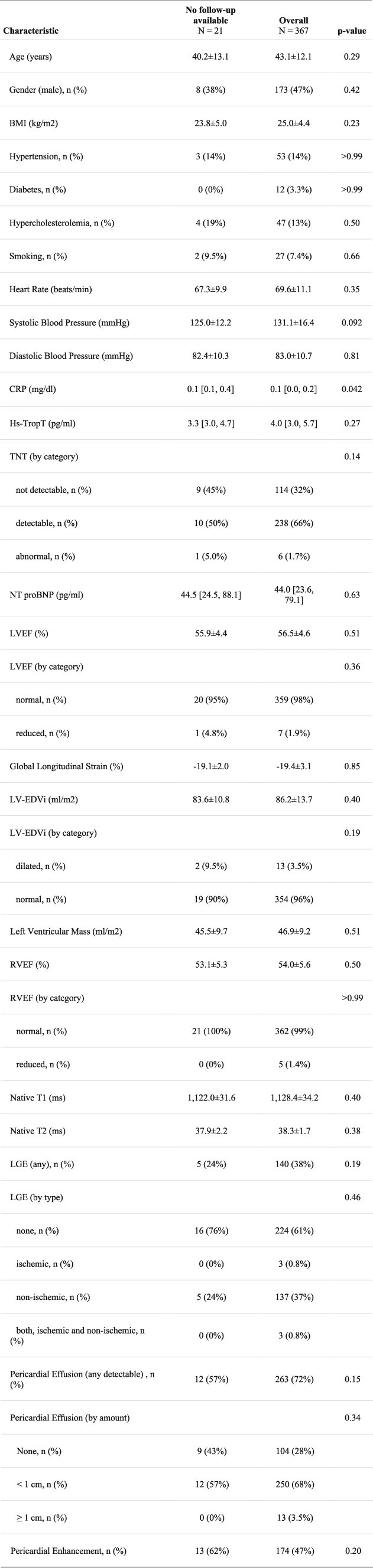
Comparison between participants without the follow-up with the whole COVID-19 cohort

Comparison between participants without the follow-up with the whole COVID-19 cohort. BMI – body mass index, BP – blood pressure, CRP – C-reactive protein; hs-TropT – high-sensitive troponin T, NT pro BNP – N terminal pro brain natriuretic peptide, LV- EDVi – Left ventricular end-diastolic volume, indexed to body surface area, LVEF – LV ejection fraction, GLS – global longitudinal strain; RVEF – right ventricular ejection fraction, LGE – late gadolinium enhancement, ms – millisecond, ml – milliliter, m – meter, bpm – beats per minute, mmHg, millimeter Mercury, pg – picogram. Ischemic and non-ischemic scar may occur in the same patient. The categorical classifications of LVEF, RVEF and LV-EDVi are based on the UK Biobank study^30^.

**Extended Data Table 2:**
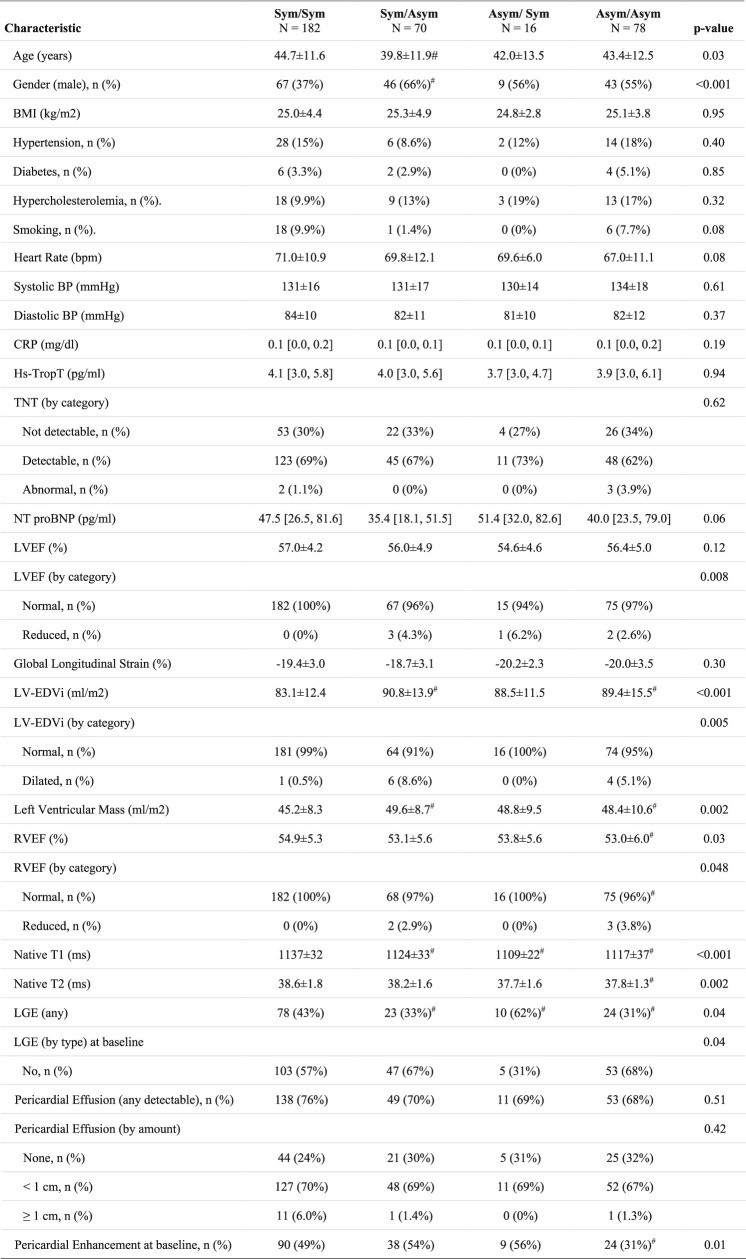
Baseline characteristics of subgroups stratified by symptom status at baseline and at follow-up

Baseline characteristics of subgroups stratified for the symptom status at baseline and at follow-up. Cardiac symptoms were present in 198 (57%) patients at follow-up; symptoms persisted in a total of 182 (53%) patients; 16 (5%) previously asymptomatic patients developed new symptoms, 78 (23%) originally asymptomatic patients remained asymptomatic, and 70 (20%) patients became asymptomatic. Sym – symptomatic symptom status, Asym – asymptomatic symptom status; Subgroup: at baseline/at follow-up. BMI – body mass index, BP – blood pressure, CRP – C-reactive protein; hs-TropT – high-sensitive troponin T, NT pro BNP – N terminal pro brain natriuretic peptide, LV- EDVi – Left ventricular end-diastolic volume, indexed to body surface area, LVEF – LV ejection fraction, GLS – global longitudinal strain; RVEF – right ventricular ejection fraction, LGE – late gadolinium enhancement, ms – millisecond, ml – milliliter, m – meter, bpm – beats per minute, mmHg, millimeter Mercury, pg – picogram. Ischemic and non-ischemic scar may occur in the same patient. The categorical classifications of LVEF, RVEF and LV-EDVi are based on the UK Biobank study^30^. Posthoc testing was performed at the level of p≤0.05 in the global analysis. Significant posthoc tests using Bonferroni correction are denoted as # vs. Sym/Sym, § Asym vs. Sym.

**Extended Data Table 3:**
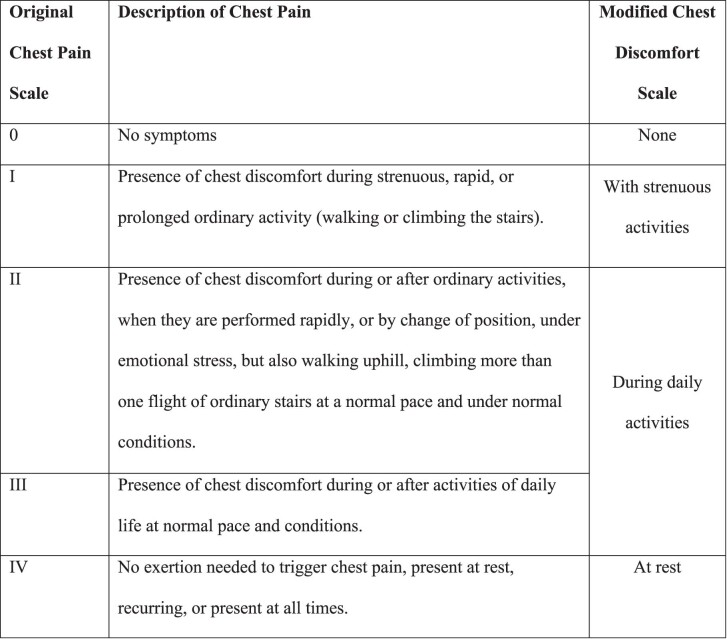
Modified Chest Discomfort Scale

**Extended Data Table 4:**
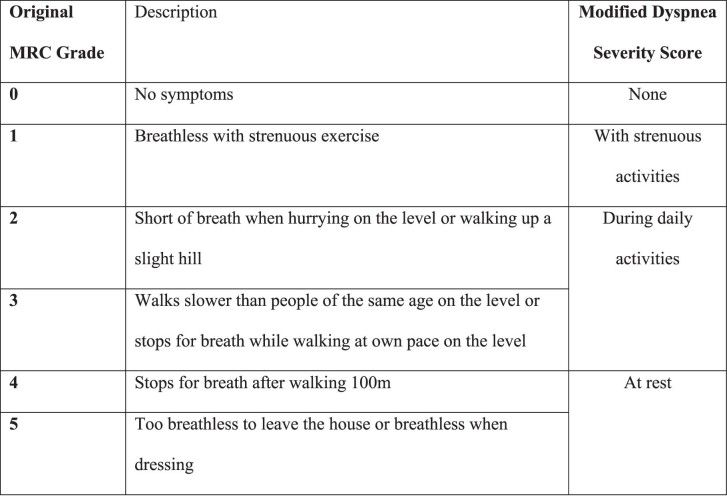
Modified Dyspnea Severity Score

**Extended Data Table 5:**
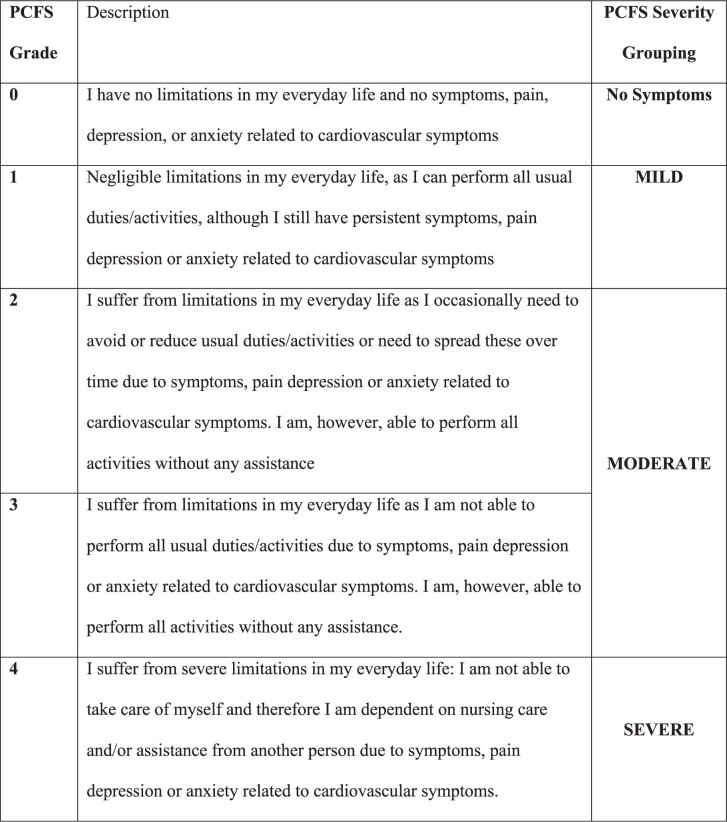
PCFS scale (modified for cardiovascular symptoms)

## Data Availability

A relatively small cohort of well-characterized patients make the risk of identification of sensitive data of individual patients possible. Also, this is an ongoing study, and completion of the outcome endpoint has not yet been attained. Therefore, the data are not openly accessible.

## Code availability

The R code used to generate all results in this paper is publicly available on GitHub (https://github.com/CovidHeart/NM2022-ImpressionCOVIDHeart).
