# Supplementary material for: Long-term cardiac pathology in individuals with mild initial COVID-19 illness
Source: Nat Med. 2022 Sep 5;28(10):2117–23. doi: 10.1038/s41591-022-02000-0 (PMC9556300; doi:10.1038/s41591-022-02000-0)
Supplement: Supplementary file 1 — Reporting Summary [file 41591_2022_2000_MOESM1_ESM.pdf]

nature research

Corresponding author(s): Dr Valentina Puntmann

Last updated by author(s): 2022/08/04

Reporting Summary

Nature Research wishes to improve the reproducibility of the work that we publish. This form provides structure for consistency and transparency in reporting. For further information on Nature Research policies, see our Editorial Policies and the Editorial Policy Checklist.

Statistics

For all statistical analyses, confirm that the following items are present in the figure legend, table legend, main text, or Methods section.

n/a	Confirmed	
☐	☒	The exact sample size (n) for each experimental group/condition, given as a discrete number and unit of measurement
☐	☒	A statement on whether measurements were taken from distinct samples or whether the same sample was measured repeatedly
☐	☒	The statistical test(s) used AND whether they are one- or two-sided<br>*Only common tests should be described solely by name; describe more complex techniques in the Methods section.*
☐	☒	A description of all covariates tested
☐	☒	A description of any assumptions or corrections, such as tests of normality and adjustment for multiple comparisons
☐	☒	A full description of the statistical parameters including central tendency (e.g. means) or other basic estimates (e.g. regression coefficient) AND variation (e.g. standard deviation) or associated estimates of uncertainty (e.g. confidence intervals)
☐	☒	For null hypothesis testing, the test statistic (e.g. F, t, r) with confidence intervals, effect sizes, degrees of freedom and P value noted<br>*Give P values as exact values whenever suitable.*
☒	☐	For Bayesian analysis, information on the choice of priors and Markov chain Monte Carlo settings
☒	☐	For hierarchical and complex designs, identification of the appropriate level for tests and full reporting of outcomes
☒	☐	Estimates of effect sizes (e.g. Cohen's d, Pearson's r), indicating how they were calculated

Our web collection on statistics for biologists contains articles on many of the points above.

Software and code

Policy information about availability of computer code

Data collection	Data collection was performed using REDCap (Research Electronic Data Capture, Vanderbilt University)
Data analysis	Data were analyzed using the R Studio Version 3.6.1; SuiteHeart; Neosoft LPC, WI, US; Medis Suite MR v2.1 (Medis medical imaging systems, Leiden, The Netherlands

For manuscripts utilizing custom algorithms or software that are central to the research but not yet described in published literature, software must be made available to editors and reviewers. We strongly encourage code deposition in a community repository (e.g. GitHub). See the Nature Research guidelines for submitting code & software for further information.

Data

Policy information about availability of data

All manuscripts must include a data availability statement. This statement should provide the following information, where applicable:

- Accession codes, unique identifiers, or web links for publicly available datasets
- A list of figures that have associated raw data
- A description of any restrictions on data availability

A relatively small cohort of well characterized patients make the risk of identification of sensitive data of individual patients possible. Also, this is an ongoing study and completion of the outcome endpoint has not yet been attained. Therefore the data are not openly accessible. The R code used to generate all results in this paper is publicly available on GitHub (https://github.com/CovidHeart/NM2022-ImpressionCOVIDHeart)

Field-specific reporting

Please select the one below that is the best fit for your research. If you are not sure, read the appropriate sections before making your selection.

☒ Life sciences ☐ Behavioural & social sciences ☐ Ecological, evolutionary & environmental sciences

For a reference copy of the document with all sections, see nature.com/documents/nr-reporting-summary-flat.pdf

Life sciences study design

All studies must disclose on these points even when the disclosure is negative.

Sample size	COVID participants 415, controls 95; This was an exploratory study in a new disease entity and the effect size was unknown, as such an a priori sample size estimation was not possible
Data exclusions	any significant cardiac or non cardiac comorbidities, or patients hospitalised during the acute COVID illness<br>1. hospitalized during the acute COVID illness<br>2. an established diagnosis of cardiovascular disease (defined as a previous diagnosis of hemodynamically significant coronary artery disease, history of revascularization or cardiovascular device implantation, or structural heart disease, heart failure, cardiomyopathy, significant valvular disease (≥ grade III), congenital heart disease, peripheral artery disease, etc.)<br>3. known preexisting significant lung conditions, or had persistently abnormal lung function test after recovery from the acute infection;<br>4. patients with known liver or kidney disease, uncontrolled diabetes, or other significant endocrine, rheumatological or oncological conditions or receiving any treatment; clinically and laboratory euthyroid patients receiving thyroxine supplements were not excluded.<br>5. patients with known absolute contraindications to MRI,<br>6. patients unwilling to participate in long-term follow ups.
Replication	N/A, no new methods development
Randomization	N/A, not part of study design
Blinding	N/A, not part of study design

Reporting for specific materials, systems and methods

We require information from authors about some types of materials, experimental systems and methods used in many studies. Here, indicate whether each material, system or method listed is relevant to your study. If you are not sure if a list item applies to your research, read the appropriate section before selecting a response.

Materials & experimental systems

n/a	Involved in the study	
☒	☐	Antibodies
☒	☐	Eukaryotic cell lines
☒	☐	Palaeontology and archaeology
☒	☐	Animals and other organisms
☐	☒	Human research participants
☐	☒	Clinical data
☒	☐	Dual use research of concern

Methods

n/a	Involved in the study	
☒	☐	ChIP-seq
☒	☐	Flow cytometry
☒	☐	MRI-based neuroimaging

Human research participants

Policy information about studies involving human research participants

Population characteristics	a prospective single-center observational cohort study of subjects with possible subclinical cardiac involvement but no formal clinical indication for CMR; people after COVID19 infection, and uninfected controls. Relevant covariates for research population include clinical meta-data (age, gender, blood pressure, heart rate, symptoms, cardiac risk factors, cardiac biomarkers (troponin T, CRP, NTpro BNP, magnetic resonance imaging of the heart (cardiac volumes, function, strain, mapping, visualization of late gadolinium enhancement)
Recruitment	Participants were informed about the study via promotional material, disseminated via family practitioners, health authority centers, patient online groups and websites. All COVID-19 participants underwent systematic screening for eligibility prior to enrollment into the study, conducted by trained clinical research personnel using a standardized questionnaire. Subjects were excluded from the study if they had previously known heart disease or notable co-morbidities, or abnormal lung function tests following COVID-19 infection.
Ethics oversight	Ethics Committee of University Hospital Frankfurt of Goethe University

Note that full information on the approval of the study protocol must also be provided in the manuscript.

Clinical data

Policy information about clinical studies

All manuscripts should comply with the ICMJE guidelines for publication of clinical research and a completed CONSORT checklist must be included with all submissions.

Clinical trial registration	Impression Study NCT04444128
Study protocol	the relevant protocol details are published on clinicaltrials.gov NCT04444128
Data collection	Study recruitment commenced in April 2020. All consenting participants attended a baseline visit for collection of clinical data, cardiac biomarkers and imaging at University Hospital Frankfurt. The baseline visit was scheduled after a minimum of 4 weeks from the diagnosis of the acute COVID-19 illness. Follow-up visit was scheduled after a minimum of 4 months from the baseline visit. At each visit, demographic characteristics, risk factors, symptoms, resting blood pressure and heart rate, blood sampling, and magnetic resonance imaging were performed in all participants.
Outcomes	this is an exploratory study in a disease entity with a possible cardiovascular relevance, we conducted analyses for relationships of imaging markers with clinical characteristics, cardiovascular risk factors, routine cardiac imaging, sequence-specific ranges in health and disease; exploratory study in a new disease entity
